# Effects of dietary trace mineral levels on physiological responses, reproductive performance, litter performance, blood profiles, and milk composition in gestating sows

**DOI:** 10.5713/ab.23.0193

**Published:** 2023-08-23

**Authors:** Hong Jun Kim, Xing Hao Jin, Sun Woo Kang, Yoo Yong Kim

**Affiliations:** 1 Department of Agricultural Biotechnology and Research Institute of Agriculture and Life Science, Seoul National University, Seoul 08826, Korea

**Keywords:** Blood Profiles, Gestating Sow, Piglets, Trace Mineral Level

## Abstract

**Objective:**

This study was conducted to evaluate the effects of optimal trace mineral levels on the physiological responses, reproductive performance, litter performance, blood profiles and milk composition in gestating sows.

**Methods:**

A total of 59 multiparous sows (Yorkshire×Landrace) with similar body weight (BW), backfat thickness (BF), and parity were assigned to one of four treatments with 14 or 15 sows per treatment using a completely randomized design. The treatments were 100% (M1), 300% (M3), 600% (M6), and 900% (M9) of the National Research Council (NRC) *Nutrient Requirements of Swine*. During lactation period, all the sows were fed the same commercial lactation diet.

**Results:**

No significant differences were observed in the BW, BF, reproductive performance, milk composition, or growth performance of the piglets. On day 70 of gestation, the serum zinc concentration showed a quadratic response to M6 treatment (quadratic, p<0.05). Moreover, as the dietary mineral levels increased, the zinc concentration increased linearly at 110 days of gestation (linear, p<0.05). Furthermore, copper and iron concentrations in the serum of sows at 24 h postpartum decreased linearly when high levels of dietary minerals were provided (linear, p<0.05). In the serum of piglets, serum zinc concentrations decreased linearly (linear, p<0.05), and iron concentration showed a quadratic response (quadratic, p<0.05) with an increase in trace mineral premix levels in gestation diets.

**Conclusion:**

The current trace mineral requirements of NRC (2012) are suitable for gestating sows, and the addition of dietary mineral levels in the gestating diet did not show any improvements during the gestation and lactation periods.

## INTRODUCTION

Pigs require macro- and micro-minerals for maintenance, growth, and reproduction [1]. Macrominerals, such as calcium, phosphorus, sodium, and chlorine, play important roles in acid-base balance, bone formation, and signal transmission [2]. Microminerals, including zinc, copper, iron, and manganese are required in smaller amounts than macrominerals, and function as cofactors and facilitators of enzymatic reactions [3]. However, an insufficient zinc intake can damage the innate immune system [4] and cause reproductive failure [5]. Copper deficiency may also lead to a critical reduction in osteoblast activity and less deposition of calcified cartilage matrix [6]; in addition, prolonged hepatic Fe mobilization without providing extra dietary iron could eventually lead to anemia in animals.

Generally, the availability of trace mineral are limited and supplemented high dose are required to fulfil animal needs which often results in toxicity issues and deficiency of other trace mineral elements [7]. As trace mineral contents in pig diets may vary according to storage temperature and humidity [8,9], this requirement is also affected by stress status or physiological conditions in animals [10]. Many studies have focused only on the effects of single trace mineral levels or mineral source in sows. Payne et al [11] demonstrated that a gestational diet containing 200 mg/kg zinc increased litter birth weight in sows. Increasing dietary copper levels (20, 120, or 220 mg/kg) linearly increased the live birth body weight (BW) of piglets and copper concentration in milk [12]. Buffler et al [13] also reported that high levels of dietary iron (256 vs 114 mg/kg) in sow feed resulted in greater iron retention and improved litter size and weight.

Dietary trace mineral interactions were importantly considered to pig diet formulation, especially copper, zinc, and iron contents. In older studies, high concentrations of dietary zinc induce the intestinal metallothionein to bind copper and increase copper requirement [14]. Moreover, high intakes of iron decrease copper absorption due to competition within the intestinal tract [15]. So, in recent little information is available regarding the effects of trace mineral premix levels and their interactions on the performance of sows and their progeny.

Therefore, the current study was conducted to investigate the effects of different levels of dietary trace mineral premix on the physiological responses, reproductive performance, serum mineral content of sows and piglets, and milk composition during the entire gestation period.

## MATERIALS AND METHODS

### Animals

All experimental procedures involving animals were conducted following the Animal Experimental Guidelines provided by the Seoul National University Institutional Animal Care and Use Committee (SNUIACUC; SNU-200128-5).

A total of 64 F1 multiparous sows (Yorkshire×Landrace) with average BW of 231.5 kg, average backfat thickness of 23.3 mm, and an average parity of 4.98 was allotted to one of four treatments considering BW, backfat thickness, and parity in completely randomized design after mating. All sows took two times of artificial insemination service according to estrus cycle after weaning and checked pregnancy at day 35 of gestation by ultrasound scanner (Donjin BLS, Gwangju, Korea). After pregnancy diagnosis, only 59 F1 multiparous sows were pregnant and continued to consume their treatment diets. During the experimental period, multiparous sows of third or over third parity were fed a 2.4 kg/d gestation diet.

### Experimental design and diet

The experiment was allotted to one of 4 treatments considering BW, backfat thickness, and parity in completely randomized design. All experimental diets for gestating sows were formulated based on corn-soybean meal and trace mineral premix was supplemented by treatment levels. The treatments were 100% (M1), 300% (M3), 600% (M6), and 900% (M9) of the NRC [16] requirements. The trace mineral premix formulas in the gestation diet are shown in Table 1. All other nutrients were formulated to meet or exceed the NRC [16] requirements. Table 2 shows the formulas and chemical compositions of gestation and lactation diet. All sows were fed the same commercial lactation diet during the lactation period.

### Animal management

All experimental sows (parity: 3 to 6) were fed an experimental diet once a day at 08:00 h and provided 2.4 kg/d during gestation and the gestation diet was decreased gradually 0.2 kg/d during 5 days before farrowing. After farrowing, sows were fed lactation diet of 1, 2, 3, 4, and 5 kg/d as lactating age increased and fed a diet *ad libitum* until weaning.

All sows were accommodated in individual gestation stalls (2.20×0.64 m) where the indoor temperature was regulated to an average of 20°C by an automatic ventilation system. At day 110 of gestation, sows were moved from the gestation barn to farrowing crates (2.50×1.80 m) after washing and disinfecting their body, especially breast and vulva. None of the sows were treated with delivery inducer and they were assisted when dystocia occurred. The room temperature of the lactating barn was kept at 28°C±2°C and the baby house under heating lamp was kept at 32°C±2°C. The air condition of the lactating barn was regulated automatically by the ventilation system and air-conditioner. After weaning, the sows were moved to a breeding barn for the next oestrus cycle.

After farrowing, piglets were cross-fostered within treatment until 24 h postpartum to balance the suckling intensity of sows with equalization of litter size, and thus to minimize any effect of initial litter size potentially affecting litter growth. Cutting of the umbilical cord and tail and castration were conducted 3 days after birth, and piglets were injected with 150 ppm Fe-dextran (Gleptosil, Alstoe, UK) injection. All piglets were not fed creep feed during the whole lactation period. Weaning was performed at approximately 24±3 d.

### Performance of sow

The BW and backfat thickness of sows were measured at mating, day 35, 70, and 110 of gestation, 24 h postpartum, and day 21 of lactation, respectively. The BW of the sow was measured by electric scale (CAS Co. Ltd., Yangju, Korea) for sow and backfat thickness was measured at the P_2_ position (mean value form both side of the last rib and 65 mm away from the backbone) by an ultrasound device (Lean Meter, Renco Corp., Minneapolis, MN, USA). Daily feed wastage was recorded during lactation and lactation feed intake was measured when measuring the BW and backfat thickness of lactating sows at day 21 of lactation. The weaning to estrus interval (WEI) of sows, as an important parameter for evaluating reproductive performance, was measured after weaning.

### Reproductive performance

After farrowing, the number of piglets for total born, stillbirth, mummy, alive piglets was recorded, and the BW of alive piglets, stillborn, and mummy was measured by electric scale (CAS Co. Ltd., Korea). When measuring the BW of piglets, ear notching was practiced for the experiment.

### Litter performance

After ear notching, cross-fostering of the piglets within the same treatment was performed until 12 h postpartum for equalizing litter size. The number and BW of piglets was measured again at day 21 of lactation to calculate litter weight, piglet weight and both weight gain.

### Blood samples

Blood collection from sows based on similar BW and backfat thickness (n = 6 for each treatment) was taken by venipuncture of the jugular vein using 10 mL disposable syringes at mating, day 35, 70, 110 of gestation, 24 h postpartum and day 21 of lactation. Blood from suckling piglets (n = 12 for each treatment) was collected from the anterior vena cava using 3 mL disposable syringes at 24 h postpartum and 5 mL disposable syringes at day 21 of lactation. All blood samples were enclosed in serum tubes (SSTII Advance; BD Vacutainer, Becton Dickinson, Plymouth, UK) as well as ethylenediaminetetraacetic acid tube (BD Vacutainer K_2_E; Becton Dickinson, UK) and centrifuged at 3,000 rpm and 4°C for 15 min (5810R; Eppendorf, Hamburg, Germany) after clotting at room temperature for 30 min. The upper liquid (serum) of the blood was separated to a microtube (Axygen, Union City, CA, USA) and stored at −20°C freezer until later analysis.

### Milk composition

Colostrum and milk samples were taken from functional mammary glands of each sow at 24 hours post-farrowing and days 21 of lactation, respectively. Injection of 5 mL of oxytocin (Komi oxytocin inj.; Komipharm International Co., Ltd., Siheung, Korea) was into the blood vessels of the sow’s ear. Colostrum and milk were collected in 50 mL conical tubes (SPL Life Sciences Co., Ltd., Pocheon, Korea) from the first and second teats. After collection, samples were stored in a freezer (−20°C) until further analysis. Proximate analysis of colostrum and milk was conducted using Milkoscan FT120 (FOSS Electric, Hillerod, Denmark).

### Analytical methods

Serum and diets were analyzed for trace mineral using the spectrofluorometric method outlined by AOAC [17]. The analyses of serum from sows and pigs were conducted at the same time.

### Statistical analysis

All collected data were carried out by least squares mean comparisons and were evaluated with the general linear model procedure of SAS [18]. Orthogonal polynomial contrasts were used to determine the linear and quadratic effects by increasing the trace mineral premix levels in gestation for all measurements of sows and piglets. Individual sows, whole litter weight, and average pig weight within litter were considered the experimental units. Differences among means were declared significant at p<0.05 and highly significant at p<0.01 and the determination of tendency for all analyses was p≥0.05 and p<0.10.

## RESULTS

### Performance of sow

The effects of dietary trace mineral levels on gestating lactating sows are shown in Table 3. Body weight, backfat thickness, lactation feed intake, and WEI of sows were not affected by the dietary trace mineral levels during gestation or on day 21 of lactation.

### Reproductive performance and litter performance

The effects of dietary trace mineral levels on the reproductive and litter performance of the sows are shown in Table 4. No detectable differences were noted in the number of totals born, born alive, or still-born piglets among sows fed diets containing different trace mineral levels. In addition, the different dietary mineral levels had no influence on total litter weight, litter birth weight, or litter weight at 21 d of lactation.

### Milk composition

The effects of dietary trace mineral levels on the chemical composition of colostrum and milk in sows are shown in Table 5. In the present study, no significant differences were noted in casein, protein, fat, total solid, solid non-fat, lactose, and free fatty acid contents between colostrum and milk.

### Serum trace mineral concentration in gestating sows

The effects of dietary trace mineral premix levels on serum zinc, copper, and iron concentrations in gestating sows are shown in Table 6. At 70 days of gestation, a quadratic response was observed in serum zinc concentration as trace mineral premix levels increased (quadratic, p<0.05). Moreover, serum zinc concentration increased linearly when sows were fed high levels of trace mineral premix at 110 d of gestation (linear, p<0.05). At 24 h postpartum, treatments with an increase in trace mineral premix levels showed a linear decrease in serum copper concentration (linear, p<0.05). In serum iron concentration, a linear decrease was observed as trace mineral premix levels increased at 24 h postpartum (linear, p<0.05), and M6 treatment with sows fed six times the trace mineral level had higher iron concentrations than other treatments at day 21 of lactation (quadratic, p<0.05).

### Serum trace mineral concentration in piglets

The effects of dietary trace mineral premix levels on the concentrations of serum zinc, copper, and iron in piglets are shown in Table 7. At 24 h postpartum, the serum zinc concentration of piglets decreased linearly when sows were fed an increase in the trace mineral premix level (linear, p<0.05). Moreover, serum iron concentration showed a quadratic response on day 21 of lactation (p<0.05).

## DISCUSSION

Several studies have evaluated the effects of single trace mineral levels on sow performance. Peter and Mahan [19] has reported that BW and back fat thickness in sows were not influenced by increasing levels of dietary copper (5 and 16 mg/kg), iron (80 and 126 mg/kg), and zinc (49 and 122 mg/kg). Roger et al [20] has also reported no significant difference in sow BW on day 28 of lactation as dietary iron (79.8 and 115.9 mg/kg) and copper (33.2 and 44 mg/kg) levels were increased. Recently, Lu et al [12] demonstrated that no differences were observed in BW or backfat thickness when gestating sows were fed three different levels of copper (20, 120, and 220 mg/kg). In agreement with the above findings, the present study indicated that an increase in maternal trace mineral premix levels did not influence the BW or backfat thickness of sows during the entire experimental period.

Normal feed intake during lactation is closely associated with sow BW loss, milk production, and WEI [21,22]. Several researchers have demonstrated that average daily feed intake and WEI are not affected by different levels of trace minerals when gestating sows are fed different levels of trace minerals [12,19]. However, trace mineral toxicities depends upon source, dietary level, duration of feeding and age of animals, and generally the maximumtolerable dietary level for swine is set at 1,000 mg/kg of zinc, 125 to 250 mg/kg of copper [14]. In this study, the calculated trace mineral contents in the M9 treatment were 90 mg/kg Cu and 900 mg/kg zinc. Although a toxic response to high levels of trace minerals did not occur, the feed cost increased linearly when an increase in the trace mineral premix level was provided.

In the current study, feeding with different trace mineral levels did not affect the reproductive or litter performance of sows. Some studies have shown no significant differences in reproductive performance and litter growth when a single mineral was added during late gestation [23,24] and lactation [25,26]. However, Holen et al [27] reported that gestating diets containing different levels of dietary zinc (125 vs 365 mg/kg) provided during late gestation did not affect reproductive performance but high levels of zinc resulted in heavier piglet birth weight and reduced pre-weaning mortality. Lu [12] also stated that increasing copper levels (20, 120, or 220 mg/kg) linearly increased live-born piglet weight when diets were supplied during gestation and lactation. Buffler et al [13] reported that the + iron group (256 mg/kg) during gestation improved litter size and weight compared to the Fe group (114 mg/kg). However, in this experiment, the dietary trace mineral complex was supplemented to the gestation diet without only a single trace mineral added, and interactions existed among iron, zinc, and copper, which may explain why an increase in dietary trace minerals could not improve reproductive performance and litter growth.

The nutrients in sow milk are especially necessary for suckling piglets, who can only obtain nutrients from milk [28], and milk composition is mainly affected by the gestation diet, which is associated with sow body reserve and catabolism [29]. However, Peter and Mahan [19] stated that milk fat content was similar between trace mineral sources and levels. The effects of different trace mineral types on sow diets showed no significant differences in protein, fat, and lactose concentrations in the colostrum and milk [30]. Therefore, these results agreed with the results of the current study, which demonstrated that milk composition was not affected by different levels of dietary minerals.

Limited information has been gathered regarding the complicated changes in serum mineral levels (zinc, copper, and iron) when gestating sows are fed different dietary mineral premix levels. Kalinowski and Chavez [31] reported that low zinc (13 mg/kg) decreased plasma zinc levels numerically on days 86, 100, and 113 of pregnancy, and significantly on days 7 and 14 of lactation. However, in the current study, serum zinc concentrations increased linearly only on day 110 of gestation as maternal zinc levels increased. Moreover, serum copper and iron levels decreased linearly as dietary zinc levels increased at 24 h postpartum, which is consistent with a report by Hill et al [32,33] who indicated that sows fed 0, 50, or 500 mg/kg zinc had lower serum zinc and higher serum copper concentrations than sows fed 5,000 mg/kg zinc. The excess zinc inhibits absorption of copper resulting in copper deficiency and also reduces directly cellular uptake of iron [34], however, high intake of iron also could reduce the absorption of zinc [35] illustrating the antagonistic effect of dietary zinc to copper and iron was stronger than that of dietary iron to zinc, which can be a reason why serum copper and iron levels were decreased and serum zinc concentration was not changed at 24 h postpartum. In contrast, van Riet et al [24] stated that zinc and copper concentrations in the plasma did not respond to increased dietary zinc level from 46.6 to 124 mg/kg in gestating sows. Generally, the plasma zinc concentration decreased from insemination to day 50 of gestation, remained stable at day 110 of gestation, and decreased until weaning.

Poulsen [36] reported no significant difference in the serum copper concentration of piglets when copper concentrations in serum and milk were increased in sows fed with high copper diet (116 vs 8 mg/kg). Feeding low zinc to sows during late gestation and lactation reduced plasma zinc levels in piglets on day 7 and significantly on day 14 of age [31]. However, in this study serum zinc in piglet was decreased linearly as maternal mineral premix increased, which got the opposite result than previous study. This decline of zinc concentration in piglet may be related with the antagonistic effect of multiple trace minerals during gestation period. Especially, zinc can be more sensitive than other minerals in antagonistic effect. Therefore, zinc deposition in fetus decreased even though the high levels of dietary trace mineral supplied to gestating sows. After day 90 of gestation, more nutrients deposition in sow will transfer to fetus for growth [37], and before farrowing nutritional balance will be kept in sow’s body pool [38]. Another reason was indirectly speculated that the interactions among trace minerals (copper, zinc, iron) occured in piglets after colostrum intake. Unfortunately, it is very difficult to explain the change of serum trace mineral concentration in piglets based on previous reference or the change of serum trace mineral concentration in sows. Finally, this study indicated that exceeding NRC [16] mineral requirement in the gestating diet could reduce serum zinc concentration in piglets.

## CONCLUSION

The current NRC [16] trace mineral requirements are sufficient for normal metabolism of gestating sows and subsequent piglet growth. Additional mineral supplementation of diet did not show any beneficial effects for gestating and lactating sows.

## Figures and Tables

**Table 1:** Formulas and concentrations of trace mineral premix in gestation diets

Items (mg/kg)	Trace mineral requirements of the NRC [16]	Concentration of trace minerals in trace mineral premix
Copper	10	10,000
Iodine	0.14	140
Iron	80	80,000
Manganese	25	25,000
Selenium	0.15	150
Zinc	100	100,000

**Table 2:** The formulas and chemical composition of the experimental gestation diet as fed basis

Item	Gestation diet^1)^				Lactation diet

	M1	M3	M6	M9	
Ingredient (%)					
Corn	76.80	76.43	75.84	75.25	73.21
SBM-46	11.98	12.04	12.13	12.23	15.36
Wheat bran	5.99	5.97	5.97	5.96	5.00
Tallow	1.78	1.91	2.11	2.31	2.79
L-lysine HCl (78 %)	0.26	0.26	0.26	0.26	0.41
DL-methionine (99 %)	0.04	0.04	0.04	0.04	0.03
Dicalcium phosphate	1.35	1.35	1.35	1.35	1.53
Limestone	1.20	1.20	1.20	1.20	1.07
Vitamin premix^2)^	0.10	0.10	0.10	0.10	0.10
Mineral premix	0.10	0.30	0.60	0.90	0.10
Choline chloride-50	0.10	0.10	0.10	0.10	0.10
Sodium chloride	0.30	0.30	0.30	0.30	0.30
Sum	100.00	100.00	100.00	100.00	100.00
Chemical composition^3)^					
ME (kcal/kg)	3,265.03	3,265.04	3,265.00	3,265.05	3,300
CP (%)	12.00	12.00	12.00	12.00	13.43
SID Lys (%)	0.74	0.74	0.74	0.74	0.96
SID Met (%)	0.23	0.23	0.23	0.23	0.26
Ca (%)	0.75	0.75	0.75	0.75	0.76
Total P (%)	0.60	0.60	0.60	0.60	0.65

SBM, soybean meal; ME, metabolizable energy; CP, crude protein; SID, standardized ileal digestibility.

1) M1, corn-SBM based diet with 1 times of trace mineral requirement in NRC [16]; M3, SBM based diet with 3 times of trace mineral requirement in NRC [16]; M6, SBM based diet with 6 times of trace mineral requirement in NRC [16]; M9, SBM based diet with 9 times of trace mineral requirement in NRC [16].

2) Provided per kg of diet: Vit. A, 4,000 IU; Vit. D3, 800 IU; Vit. E, 44 IU; Vit. K3, 0.5 mg; Biotin, 0.20 mg; Folacin, 1.30 mg; Niacin, 10.00 mg; Calcium d-pantothenate, 12.00 mg; Rivoflavin, 3.75 mg; Thiamin 1.00 mg; Vit. B6, 1.00 mg; Vit. B12, 15.00 μg.

3) Calculated value.

**Table 3:** Effects of dietary trace mineral levels on body weight, backfat thickness, weaning to estrus interval and daily feed intake of gestating-lactating sows

Criteria	Treatment^1)^				SEM	p-value^2)^	
	
	M1	M3	M6	M9		Lin	Quad
No. of sows	16	15	17	15	-	-	-
Body weight (kg)							
At mating	231.57	234.17	228.83	231.53	3.591	0.921	0.872
Day 35	247.00	250.46	246.58	246.86	3.424	0.782	0.461
Day 35	244.77	249.79	247.81	241.70	2.923	0.321	0.081
Day 35	268.99	272.41	267.77	263.95	2.892	0.240	0.223
Changes (0 to 110 d)	−37.42	−38.24	−38.94	−32.42	2.091	0.414	0.991
24 h postpartum	252.98	257.01	247.68	251.21	3.218	0.609	0.904
Day 21 of lactation	252.93	246.89	245.96	240.82	3.542	0.277	0.919
Changes (0 to 21 d)	−0.05	−10.12	−1.72	−10.39	1.855	0.222	0.976
Backfat thickness (mm)							
At mating	23.03	23.83	23.57	22.82	0.628	0.761	0.597
Day 35	23.97	25.14	24.50	24.36	0.719	0.917	0.604
Day 70	24.00	25.25	22.62	23.15	0.726	0.390	0.791
Day 110	24.43	25.75	24.46	25.55	0.711	0.931	0.813
Changes (0 to 110 d)	−1.40	−1.92	−0.89	−2.73	0.743	0.462	0.094
24 h postpartum	23.07	25.39	22.85	23.00	0.718	0.624	0.603
Day 21 of lactation	23.07	23.11	22.69	21.80	0.694	0.514	0.794
Changes (0 to 21 d)	0.00	−2.29	−0.15	−1.20	0.584	0.861	0.751
ADFI (kg/d)	6.38	6.44	6.53	6.63	0.150	0.573	0.991
WEI (d)	3.77	3.50	3.83	4.00	0.171	0.559	0.651

SEM, standard of error of means; ADFI, average daily feed intake; WEI, weaning to estrus interval; SBM, soybean meal.

1) M1, corn-SBM based diet with 1 times of trace mineral requirement in NRC [16]; M3, SBM based diet with 3 times of trace mineral requirement in NRC [16]; M6, SBM based diet with 6 times of trace mineral requirement in NRC [16]; M9, SBM based diet with 9 times of trace mineral requirement in NRC [16].

2) Lin, linear; Quad, quadratic.

**Table 4:** Effects of dietary trace mineral levels of gestating sows on reproductive and litter performance of its progeny

Criteria	Treatment^1)^				SEM	p-value^2)^	
	
	M1	M3	M6	M9		Lin	Quad
No. of sows	16	15	17	15	-	-	-
Reproductive performance (N)							
Total born/litter	13.47	13.14	14.31	12.20	0.371	0.421	0.188
No. of born alive	12.27	12.07	12.85	11.20	0.305	0.370	0.211
No. of stillbirths	1.20	0.93	1.46	1.00	0.200	0.610	0.961
After cross-foster^3)^	12.27	12.00	12.54	11.20	0.158	0.124	0.114
Day 21 of lactation	10.93	10.43	10.92	10.30	0.162	0.376	0.753
Litter weight (kg)							
Total litter weight	18.44	17.33	19.71	18.09	0.454	0.720	0.569
Litter birth weight	16.75	16.46	17.99	17.01	0.438	0.580	0.562
After cross-foster^3)^	16.75	16.90	17.60	17.01	0.365	0.691	0.551
Day 21 of lactation	67.08	64.17	65.92	65.52	1.392	0.873	0.712
Litter weight gain	50.33	47.27	48.32	48.52	1.356	0.784	0.592
Piglet weight (kg)							
Piglet birth weight	1.37	1.40	1.41	1.53	0.033	0.110	0.588
After cross-foster^3)^	1.37	1.40	1.41	1.53	0.033	0.110	0.588
Day 21 of lactation	6.14	6.14	6.02	6.40	0.102	0.481	0.362
Piglet weight gain	4.73	4.75	4.62	4.87	0.097	0.750	0.521

SEM, standard of error of means; SBM, soybean meal.

1) M1, corn-SBM based diet with 1 times of trace mineral requirement in NRC [16]; M3, SBM based diet with 3 times of trace mineral requirement in NRC [16]; M6, SBM based diet with 6 times of trace mineral requirement in NRC [16]; M9, SBM based diet with 9 times of trace mineral requirement in NRC [16].

2) Lin, linear; Quad, quadratic.

3) After cross-fostering day at day 1 postpartum.

**Table 5:** Effects of dietary trace mineral levels of gestating sows on chemical compositions on colostrum and milk of sows

Criteria	Treatment^1)^				SEM	p-value^2)^	
	
	M1	M3	M6	M9		Lin	Quad
Protein							
24 h postpartum	10.35	8.20	8.92	8.92	0.745	0.535	0.990
Day 21 of lactation	5.01	4.92	5.00	4.98	0.077	0.801	0.794
Fat							
24 h postpartum	5.20	6.28	4.44	5.52	0.381	0.835	0.611
Day 21 of lactation	6.07	7.01	6.56	6.18	0.315	0.375	0.433
Total solid							
24 h postpartum	21.74	20.49	19.42	20.62	0.707	0.531	0.742
Day 21 of lactation	18.09	18.93	18.60	18.30	0.379	0.483	0.741
SNF							
24 h postpartum	15.51	13.13	14.04	14.06	0.746	0.600	0.940
Day 21 of lactation	11.44	11.24	11.42	11.53	0.060	0.841	0.781
Lactose							
24 h postpartum	3.82	3.97	3.90	3.97	0.118	0.314	0.754
Day 21 of lactation	5.51	5.53	5.59	5.73	0.046	0.983	0.455
FPD							
24 h postpartum	0.97	0.85	0.86	0.88	0.038	0.523	0.891
Day 21 of lactation	0.78	0.80	0.79	0.79	0.009	0.242	0.160

SEM, standard of error of means; SNF, solid not fat; FPD, freezing point depression; SBM, soybean meal.

1) M1, corn-SBM based diet with 1 times of trace mineral requirement in NRC [16]; M3, SBM based diet with 3 times of trace mineral requirement in NRC [16]; M6, SBM based diet with 6 times of trace mineral requirement in NRC [16]; M9, SBM based diet with 9 times of trace mineral requirement in NRC [16].

2) Lin, linear; Quad, quadratic.

**Table 6:** Effects of dietary trace mineral levels on serum trace mineral concentration of gestating and lactating sows

Criteria	Treatments^1)^				SEM	p-value^2)^	
	
	M1	M3	M6	M9		Lin	Quad
Zn (mg/dL)							
Initial	-------------------------------------------- 0.67 --------------------------------------------				-	-	-
35 d	0.36	0.63	0.82	0.93	0.129	0.151	0.694
70 d	1.43	0.91	2.89	1.06	0.241	0.532	0.011
110 d	0.48	1.59	2.14	2.16	0.299	0.051	0.251
24 h postpartum	0.60	1.49	0.78	0.54	0.186	0.451	0.204
Day 21 of lactation	0.84	0.54	0.50	1.08	0.136	0.500	0.149
Cu (mg/dL)							
Initial	-------------------------------------------- 1.25 --------------------------------------------				-	-	-
35 d	1.84	1.72	1.59	1.76	0.076	0.658	0.340
70 d	1.99	1.54	1.92	1.64	0.079	0.381	0.756
110 d	1.73	1.95	1.72	1.72	0.088	0.712	0.649
24 h postpartum	1.68	1.72	1.39	1.35	0.075	0.054	0.982
Day 21 of lactation	1.28	1.57	1.30	1.65	0.076	0.231	0.711
Fe (mg/dL)							
Initial	-------------------------------------------- 2.15 --------------------------------------------				-	-	-
35 d	1.94	1.11	3.09	2.08	0.241	0.070	0.272
70 d	2.73	2.50	2.55	2.12	0.202	0.441	0.841
110 d	2.69	2.56	4.19	3.32	0.390	0.342	0.494
24 h postpartum	2.64	2.63	1.12	1.04	0.277	0.013	0.693
Day 21 of lactation	0.92	0.96	2.29	1.32	0.199	0.059	0.032

SEM, standard of error of means; SBM, soybean meal.

1) M1, corn-SBM based diet with 1 times of trace mineral requirement in NRC [16]; M3, SBM based diet with 3 times of trace mineral requirement in NRC [16]; M6, SBM based diet with 6 times of trace mineral requirement in NRC [16]; M9, SBM based diet with 9 times of trace mineral requirement in NRC [16].

2) Lin, linear; Quad, quadratic.

**Table 7:** Effects of dietary trace mineral levels on serum trace mineral concentration of piglets

Criteria	Treatments^1)^				SEM	p-value^2)^	
	
	M1	M3	M6	M9		Lin	Quad
Zn (mg/dL)							
24 h postpartum	2.04	0.75	0.66	0.15	0.236	<0.001	0.211
Day 21 of lactation	0.84	0.82	0.48	0.96	0.086	0.930	0.110
Cu (mg/dL)							
24 h postpartum	0.50	0.43	0.37	0.29	0.048	0.144	0.966
Day 21 of lactation	1.32	1.63	1.46	1.43	0.088	0.921	0.439
Fe (mg/dL)							
24 h postpartum	1.98	1.91	0.96	1.14	0.281	0.210	0.675
Day 21 of lactation	1.37	4.57	2.77	2.47	0.417	0.812	0.022

SEM, standard of error of means; SBM, soybean meal.

1) M1, corn-SBM based diet with 1 times of trace mineral requirement in NRC [16]; M3, SBM based diet with 3 times of trace mineral requirement in NRC [16]; M6, SBM based diet with 6 times of trace mineral requirement in NRC [16]; M9, SBM based diet with 9 times of trace mineral requirement in NRC [16].

2) Lin, linear; Quad, quadratic.
